# New Spanish *Dinotrema* species with propodeal areola or mainly sculptured propodeum (Hymenoptera, Braconidae, Alysiinae)

**DOI:** 10.3897/zookeys.297.5228

**Published:** 2013-05-08

**Authors:** Francisco Javier Peris-Felipo, Sergey A. Belokobylskij, Ricardo Jiménez-Peydró

**Affiliations:** 1 Laboratory of Entomology and Pest Control, Institute Cavanilles of Biodiversity and Evolutional Biology, University of Valencia, c/.Catedrático José Beltrán nº2, 46980 Paterna, Valencia, Spain; 2 Museum and Institute of Zoology Polish Academy of Sciences, Wilcza 64, Warszawa 00-679, Poland; Zoological Institute Russian Academy of Sciences, St. Petersburg, 199034, Russia

**Keywords:** Braconidae, Alysiinae, *Dinotrema*, Diptera parasitoids, new species, Spain

## Abstract

The illustrated descriptions of eight new species of the genus *Dinotrema* with either the propodeum mainly sculptured or having a large propodeal areola from Spain are provided, viz. *Dinotrema amparoae* **sp. n.**, *Dinotrema benifassaense* **sp. n.**, *Dinotrema lagunasense* **sp. n.**, *Dinotrema pilarae* **sp. n.**, *Dinotrema robertoi* **sp. n.**, *Dinotrema teresae* **sp. n.,***Dinotrema tinencaense* **sp. n.,** and *Dinotrema torreviejaense* **sp. n.**.

## Introduction

The genus *Dinotrema* Foerster, 1862 is the largest braconid genus of the subfamily Alysiinae with approximately 320 species worldwide (Yu et al. 2012). The current position of the genus *Dinotrema* was suggested by van Achterberg (1988), and it differs from the related genus *Aspilota* Foerster, 1862 by the small size of the paraclypeal fovea which are distinctly separated from the inner margin of eyes. Species of *Dinotrema* parasitise in the larvae of Diptera predominantly belonging to the family Phoridae (van Achterberg 1988). Some species were also reared from the members of the families Anthomyiidae and Platypezidae (Fischer et al. 2008), but these data need to be verified.

Fischer (1972) and Tobias (2003, 2004a, 2004b, 2006) suggested division of the *Dinotrema* species into some morphological groups characterized by extend and type of sculpture of the propodeum and presence/absence of the mesoscutal pit. These features were also used in recent papers on the Western Palaearctic species of the *Dinotrema* species and in the study of its Spanish species.

In this paper, eight new Spanish species of the genus *Dinotrema* with a largely sculptured propodeum or having a large areola on the propodeum are described and illustrated, viz. *Dinotrema amparoae* sp. n., *Dinotrema benifassaense* sp. n., *Dinotrema lagunasense* sp. n., *Dinotrema pilarae* sp. n., *Dinotrema robertoi* sp. n., *Dinotrema teresae* sp. n., *Dinotrema tinencaense* sp. n., and *Dinotrema torreviejaense* sp. n.

## Material and methods

The specimens for this study were collected by Malaise traps in the Natural Parks of Carrascal de la Font Roja (Alicante Province, Spain), La Tinença de Benifassà (Castellon Province, Spain) and Las Lagunas de La Mata-Torrevieja (Alicante Province, Spain). The climatic and orographic characteristics of these parks were described by Peris-Felipo and Jiménez-Peydró (2012).

For the terminology of the morphological features and sculpture, measurements and wing venation nomenclature, see Fischer (1973). The following abbreviations are used in the paper: POL - post-ocellar line; OOL - ocular-ocellar line; OD - maximum diameter of lateral ocellus.

The types of the described species are deposited in the following entomological collections:

**ENV** Entomological collection of the University of Valencia (Spain).

**HNHM** Hungarian National History Museum, Budapest (Hungary).

**NHMW** Naturhistorisches Museum, Wien (Austria).

**BMNH** Natural History Museum, London (U.K.).

**ZISP** Zoological Institute of the Russian Academy of Sciences, St. Petersburg (Russia).

## Taxonomic part

### Dinotrema amparoae

Peris-Felipo sp. n.

urn:lsid:zoobank.org:act:31961B94-0D7F-48DF-85B2-A2AFF98B3138

http://species-id.net/wiki/Dinotrema_amparoae

Figs 1 –12

#### Type material.

Holotype: 1 female (ENV), “Spain, Alicante Province, Torrevieja, Natural Park of Lagunas de La Mata-Torrevieja, 08.ii.2005, F.J. Peris-Felipo”. Paratypes: 1 female, (ENV) same label as holotype but 04.iii.2005; 1 female (ENV), same label as holotype but 05.iv.2005; 1 female (ZISP), same label as holotype but 28.iii.2006; 1 male (ENV), same label as in holotype but 30.xi.2004.

#### Diagnosis.

This new species resembles *Dinotrema flagelliforme* (Fischer), *Dinotrema paludellae* Munk and Peris-Felipo and *Dinotrema haeselbarthi* Munk and Peris-Felipo. The new species differs from *Dinotrema flagelliforme* in having the first flagellar segment 3.1 times as long as wide (twice in *Dinotrema flagelliforme*), first metasomal tergite 1.5 times as long as its apical width (2.5 times in *Dinotrema flagelliforme*), and mesoscutal pit oval (elongate in *Dinotrema flagelliforme*). On the other hand, *Dinotrema amparoae* sp. n. differs from *Dinotrema paludellae* in having the mandible 1.2 times as long as wide (1.9 times in *Dinotrema paludellae*), first flagellar segment 3.1 times as long as wide (2.3 times in *Dinotrema paludellae*), middle flagellar segments 1.4–2.0 times as long as their width (1.1 times in *Dinotrema paludellae*), and first metasomal tergite 1.5 times as long as its apical width (2.2 times in *Dinotrema paludellae*). Finally, the new species differs from *Dinotrema haeselbarthi* in having the mandible widened towards its apex and 1.2 times as long as wide (weakly widened towards apex and 1.5 times as long as wide in *Dinotrema haeselbarthi*), hind femur 3.6 times as long as its maximum width (4.0 times in *Dinotrema haeselbarthi*), first metasomal tergite 1.5 times as long as its apical width and finely striated in its apical half (twice and distinctly striate in its apical half in *Dinotrema haeselbarthi*), and precoxal sulcus not reaching anterior margin of mesopleuron (reaching anterior margin of mesopleuron in *Dinotrema haeselbarthi*).

#### Description.

Holotype, female, length of body 2.5 mm; fore wing 2.3 mm.

*Head*. In dorsal view, 1.7 times as wide as median length, 1.5 times as wide as mesoscutum, smooth, with rounded temples behind eye. Eye in lateral view 1.6 times as high as wide and 0.9 times as wide as temple. POL 3.2 times OD; OOL 3.5 times OD. Face 1.6 times as wide as high; inner margins of eyes subparallel. Clypeus about 3.0 times as wide as high, slightly curved ventrally. Paraclypeal fovea elongated, reaching the middle distance between clypeus and eye. Mandible widened towards apex, 1.2 times as long as its maximum width. Upper tooth of mandible the longest, wider than lower tooth; middle tooth wide basally, narrowed towards apex, rounded apically; lower tooth short, rounded apically. Antennae thick, 17–18-segmented. Scape twice as long as pedicel. First flagellar segment 3.1 times as long as its apical width, 1.1 times as long as second segment; second segment 1.9 times as long as its maximum width. Third to ninth flagellar segments 1.9–2.0 times as long as their width; tenth to fifteenth segments 1.4–1.5 times as long as their width; sixteenth segment 1.8–1.9 times as long as its width.

*Mesosoma*. In lateral view, as long as high. Mesoscutum 1.2 times as long as maximum width. Notauli largely absent. Mesoscutal pit present and rounded. Prescutellar depression smooth, without lateral carinae. Precoxal sulcus (= “sternaulus” of some authors) present, not reaching anterior and posterior margins of mesopleuron. Posterior mesopleural furrow smooth. Propodeum sculptured, with median longitudinal carina crossing from anterior to posterior margin, with numerous emerging lateral anastomose carinae and rugosity reaching propodeal edges. Propodeal spiracles small.

*Legs*. Hind femur 3.6 times as long as wide. Hind tibia weakly widened to apex, 10.3 times as long as its maximum subapical width, 0.9 times as long as hind tarsus. First segment of hind tarsus about twice as long as second segment.

*Wings*. Length of fore wing 2.7 times its maximum width. Vein r1 present. Radial cell reaching apex of wing, 4.0 times as long as its maximum width. Nervulus weakly postfurcal. Brachial cell closed, short, widened apically, 2.5 times as long as its maximum width. Hind wing 5.4 times as long as its maximum width.

*Metasoma*. Distinctly compressed. First tergite weakly widened towards apex, 1.6 times as long as its apical width, its apical half finely and sparsely striate. Ovipositor twice as long as first tergite, distinctly shorter than metasoma, 1.1 times as long as hind femur.

*Colour*. Body and legs brown to dark brown. Wings hyaline. Pterostigma brown.

*Male*. Body 1.8–1.9 mm; fore wing 2.0–2.1 mm. Antenna more than 17-segmented (apical segments missing). First flagellar segment 3.5 times, and second flagellar segment 3.0 times as long as wide. Third to fifteenth flagellar segment 2.6–2.7 times as long as their width. Otherwise similar to female.

**Figures 1–6.**
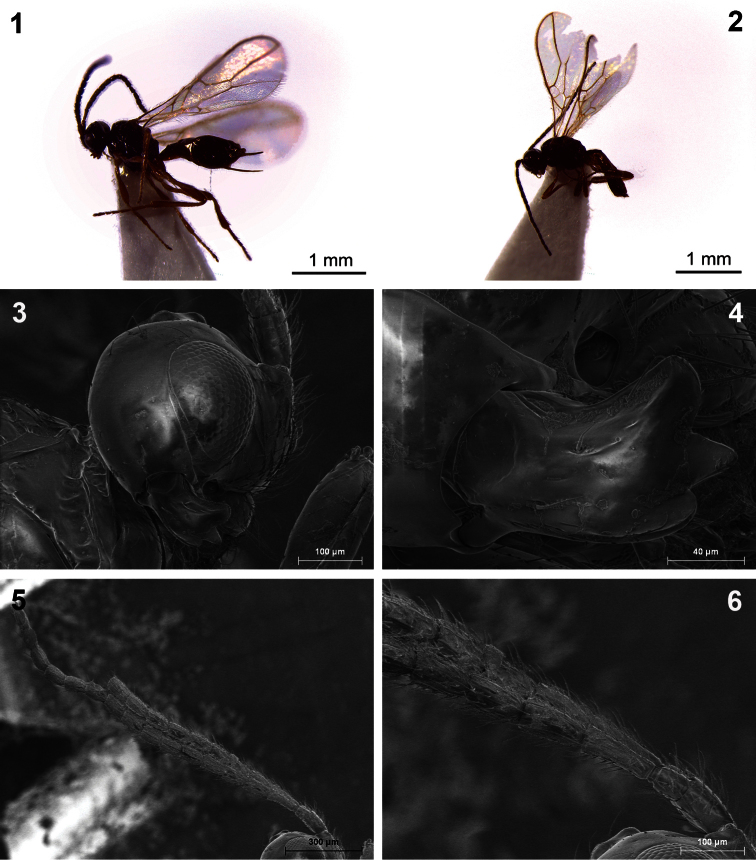
*Dinotrema amparoae* sp. n. (**1, 3–6** female; **2** male). **1, 2** Habitus, lateral view **3** Head, lateral view **4** Mandible **5** Antenna **6** Basal segments of antenna.

**Figures 7–12.**
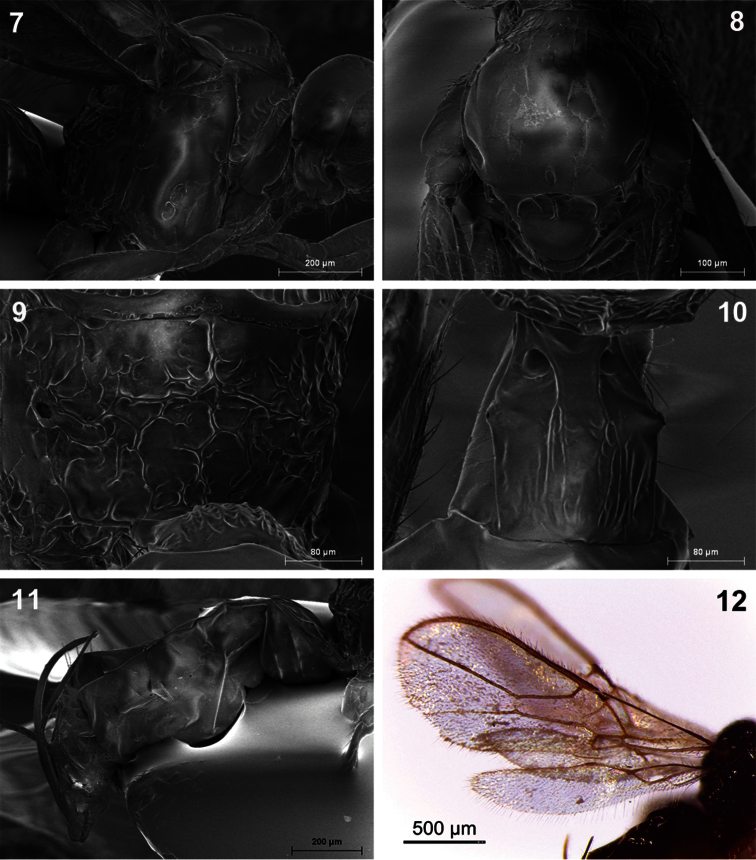
*Dinotrema amparoae* sp. n. (female).**7** Mesosoma **8** Mesonotum **9** Propodeum **10** First metasomal tergite **11** Metasoma and ovipositor **12** Fore and hind wings.

#### Etymology.

Named in honour of Amparo Felipo for her help, support and patience during my work on the PhD thesis.

### Dinotrema benifassaense

Peris-Felipo sp. n.

urn:lsid:zoobank.org:act:6BE1853F-1EE2-45BF-AA3C-2D59811ED941

http://species-id.net/wiki/Dinotrema_benifassaense

Figs 13 –24

#### Type material.

Holotype: 1 female (ENV), “Spain, Castellon Province, Pobla de Benifassà, Natural Park of Tinença de Benifassà, 11.vi.2007, F.J. Peris-Felipo”. Paratype: 1 female (ENV), same label as holotype but 14.v.2007.

#### Diagnosis.

This new species resembles *Dinotrema perlustrandum* (Fischer) and *Dinotrema concinnum* (Haliday). *Dinotrema benifassaense* sp. n. differs from *Dinotrema perlustrandum* in having the first flagellar segment 1.9 times as long as wide (3.0 times in *Dinotrema perlustrandum*), middle flagellar segments as long as their width (1.7 times in *Dinotrema perlustrandum*), mandible 0.8 times as long as wide (1.7 times in *Dinotrema perlustrandum*), and first metasomal tergite 1.8 times as long as its apical width (2.3 times in *Dinotrema perlustrandum*). On the other hand, *Dinotrema benifassaense* differs from *Dinotrema concinnum* in having the first metasomal tergite almost smooth in its apical half and 1.8 times as long as its apical width (striate in apical half and 1.9 times in *Dinotrema concinnum*) and middle flagellar segments as long as their width (1.3 times in *Dinotrema concinnum*).

#### Description.

Holotype, female, length of body 2.1 mm; fore wing 2.0 mm.

*Head*. In dorsal view, 1.6 times as wide as median length, 1.5 times as wide as mesoscutum, smooth, with rounded temples behind eye. Eye in lateral view 1.6 times as high as wide and 0.8 times as wide as temple. POL 2.8 times OD; OOL 2.5 times OD. Face 1.4 times as wide as high; inner margins of eyes subparallel. Clypeus 3.0 times as wide as high, slightly curved ventrally. Paraclypeal fovea elongate, reaching middle of distance between clypeus and eye. Mandible widened towards apex, 0.8–0.9 times as long as its maximum width. Upper tooth of mandible large, wider than lower tooth; middle tooth the longest, wide basally and narrowed towards apex, pointed apically; lower tooth short, rounded apically. Antenna thick, 17-segmented. Scape 1.8 times as long as pedicel. First flagellar segment 1.9 times as long as its apical width, 1.3 times as long as second segment; second segment 1.2 times as long as its maximum width. Third to fourteenth flagellar segments about as long as their width, fifteenth segment twice as long as its width.

*Mesosoma*. In lateral view, 1.1 times as long as high. Mesoscutum as long as maximum width. Notauli mainly absent. Mesoscutal pit present and oval. Prescutellar depression smooth, with lateral carinae. Precoxal sulcus (precoxal suture) present, not reaching anterior and posterior margins of mesopleuron. Posterior mesopleural furrow crenulate below. Propodeum sculptured, with short basal median longitudinal carina, with emerging long and undulate lateral carinae reaching propodeal edges and rugosity. Propodeal spiracles small.

*Legs*. Hind femur 3.4 times as long as wide. Hind tibia weakly widened to apex, about 8.6 times as long as its maximum sub-apical width, 0.9 times as long as hind tarsus. First segment of hind tarsus 1.9 times as long as second segment.

*Wings*. Length of fore wing 2.4 times its maximum width. Vein r1 present. Radial cell reaching to apex of wing, 3.8 times as long as its maximum width. Nervulus weakly postfurcal. Brachial cell closed, short, widened apically, 2.8 times as long as its maximum width. Hind wing 8.2 times as long as its maximum width.

*Metasoma*. Distinctly compressed. First tergite weakly widened towards apex, 1.8 times as long as its apical width, almost smooth on wide median area and striate laterally. Ovipositor 1.5 times as long as first tergite, distinctly shorter than metasoma, 1.0 times as long as hind femur.

*Colour*. Body and legs brown to dark brown. Wings hyaline. Pterostigma brown.

Male. Unknown.

**Figures 13–18.**
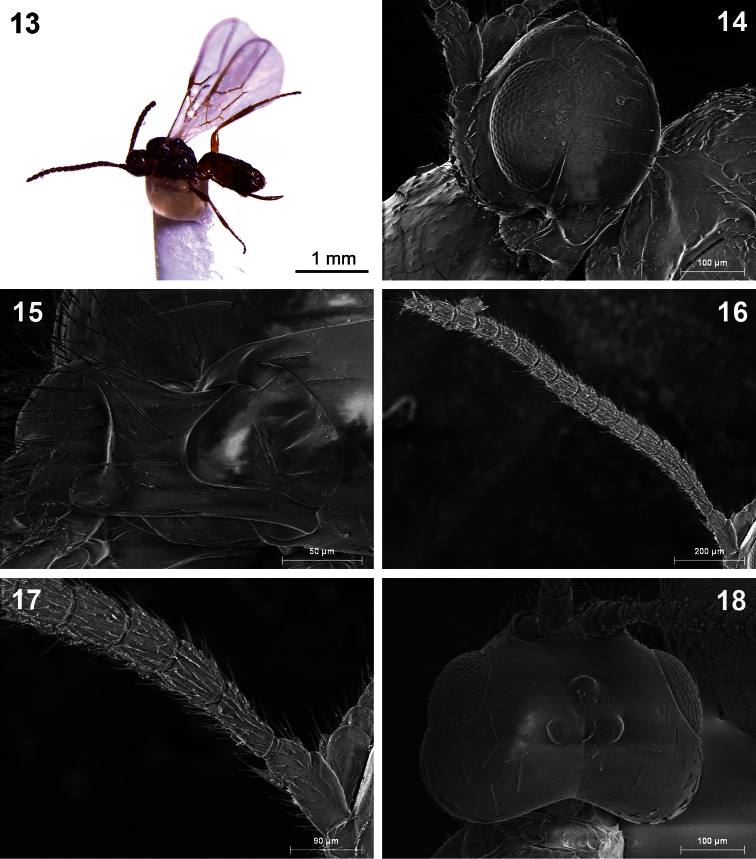
*Dinotrema benifassaense* sp. n. (female). **13** Habitus, lateral view **14** Head, lateral view **15** Mandible **16** Antenna **17** Basal segments of antenna **18** Head, dorsal view.

**Figures 19–24.**
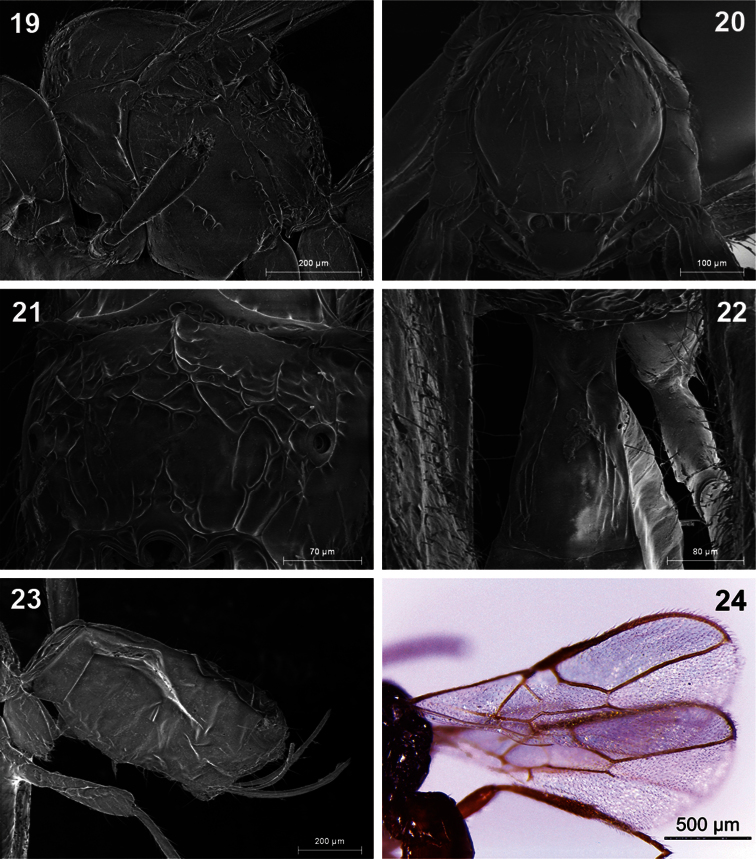
*Dinotrema benifassaense* sp. n. (female).**19** Mesosoma **20** Mesonotum **21** Propodeum **22** First metasomal tergite **23** Metasoma and ovipositor **24** Fore wing.

#### Etymology.

The name is referring to the type locality of the new species, Natural Park of Pobla de Benifassà in Castellon (Spain).

### Dinotrema lagunasense

Peris-Felipo sp. n.

urn:lsid:zoobank.org:act:20AEDC60-1D5F-4043-B79E-D6A0E3B19A4A

http://species-id.net/wiki/Dinotrema_lagunasense

Figs 25 –36

#### Type material.

Holotype: 1 female (ENV), “Spain, Alicante Province, Torrevieja, Natural Park of Lagunas de La Mata-Torrevieja, 23.iii.2005, F.J. Peris-Felipo”. Paratypes: 1 male (ENV), same label as holotype but 16.xi.2004; 1 female and 1 male (ENV), same label as holotype but 30.xi.2004; 2 females and 2 males (ENV), same label as holotype but 30.xi.2004; 3 females (ENV), same label as holotype but 18.i.2005; 1 male (ENV), same label as holotype but 26.i.2005; 1 male (ENV), same label as holotype but 15.ii.2005; 1 female (ENV), same label as holotype but 04.iii.2005; 1 male (ENV), same label as holotype but 18.iii.2005; 4 males (ENV), same label as holotype but 23.iii.2005; 7 females and 3 males (ENV), same label as holotype but 29.iii.2005; 1 female and 1 male (ENV), same label as holotype but 05.iv.2005; 1 female and 1 male (ENV), same label as holotype but 26.iv.2005; 1 male (ENV), same label as holotype but 01.xi.2005; 1 male (ENV), same label as holotype but 15.xi.2005; 4 females (ENV), same label as holotype but 02.xii.2005; 1 female (ENV), same label as holotype but 12.xii.2005; 2 females (ENV), same label as holotype but 27.xii.2005; 1 male (ENV), same label as holotype but 31.i.2006; 1 female and 1 male (ENV), same label as holotype but 07.ii.2006; 1 male (ENV), same label as holotype but 14.iii.2006; 3 males (ENV), same label as holotype but 28.iii.2006; 2 males (ENV), same label as holotype but 23.v.2006; 4 females and 2 males (ENV), same label as holotype but 28.xi.2006; 5 females (ENV), same label as holotype but 05.xii.2006; 2 males (ENV), same label as holotype but 05.xii.2006; 1 male (ENV), same label as holotype but 20.iii.2007; 1 male (ENV), same label as holotype but 15.v.2007; 1 male (ENV), same label as holotype but 30.x.2007; 1 female and 1 male (ENV), same label as holotype but 06.xi.2007; 1 female (ZISP), same label as holotype but 02.ii.2005; 1 female (ZISP), same label as holotype but 23.iii.2005; 1 female and 2 males (ZISP), same label as holotype but 29.iii.2005; 1 male (NHMW), same label as holotype but 05.iv.2005; 1 female (NHMW), same label as in holotype but 01.xi.2005; 1 female (BMNH), same label as holotype but 15.xi.2005; 1 male (BMNH), same label as holotype but 29.xi.2005; 1 male (HNHM), same label as holotype but 04.iv.2006; 1 female (HNHM), same label as holotype but 05.xii.2006.

#### Diagnosis.

This new species resembles *Dinotrema adventum* (Fischer) (comb. n.), *Dinotrema sternaulicum* (Fischer) and *Dinotrema torreviejaense* Peris-Felipo sp. n. The new species differs from *Dinotrema adventum* in having the mandible as long as wide (1.5 times in *Dinotrema adventum*), first flagellar segment 3.3 times as long as wide (twice in *Dinotrema adventum*), and precoxal sulcus not reaching anterior margin of mesopleuron (reaching in *Dinotrema adventum*). On the other hand, *Dinotrema lagunasense* sp. n. differs from *Dinotrema sternaulicum* in having the mandible as long as wide (1.4 times in *Dinotrema sternaulicum*), hind femur 3.9–4.0 times as long as its maximum width (3.5 times in *Dinotrema sternaulicum*), middle flagellar segments 1.8–2.0 times as long as their width (1.5 times in *Dinotrema sternaulicum*) and mesoscutal pit rounded (elongate in *Dinotrema sternaulicum*). Finally, new species differs from *Dinotrema torreviejaense* in having the first metasomal tergite 1.8 times as long as its apical width and striate in apical half (2.3 times as long as its apical width and smooth in *Dinotrema torreviejaense*), hind femur 3.9 times as long as its maximum width (3.6 times in *Dinotrema torreviejaense*), and mesoscutal pit rounded (oval in *Dinotrema torreviejaense*).

#### Description.

Holotype, female, length of body 1.9 mm; fore wing 1.9 mm.

*Head*. In dorsal view, nearly twice as wide as median length, 1.3 times as wide as mesoscutum, smooth, with rounded temples behind eye. Eye in lateral view about 1.5 times as high as wide and 1.2 times as wide as temple. POL 3.5 times OD; OOL 3.4 times OD. Face 1.8 times as wide as high; inner margins of eyes subparallel. Clypeus 3.0 times as wide as high, slightly curved ventrally. Paraclypeal fovea elongate, reaching middle of distance between clypeus and eye. Mandible widened towards apex, as long as its maximum width. Upper tooth of mandible large; middle tooth the longest, wide basally and narrowed towards apex, almost rounded apically; lower tooth short and wide, wider than upper tooth, rounded apically. Antenna thick, 13–16-segmented. Scape 1.5 times as long as pedicel. First flagellar segment 3.0 times as long as its apical width, 1.2 times as long as second segment; second segment 2.5 times as long as its maximum width. Third to sixth flagellar segments about 1.6 times as long as their width, seventh to fifteenth segments 1.8 times as long as their width, sixteenth flagellar segment 2.5 times as long as its width.

*Mesosoma*. In lateral view, 1.3 times as long as high. Mesoscutum as long as maximum width. Notauli largely absent. Mesoscutal pit present and rounded. Prescutellar depression smooth, without lateral carinae. Precoxal sulcus present, not reaching anterior and posterior margins of mesopleuron. Posterior mesopleural furrow smooth below. Propodeum sculptured, with more or less pentagonal areola crossed by median longitudinal carina, areola margins sculptured with emerging carinae reaching propodeal margins. Propodeal spiracles relatively small.

*Legs*. Hind femur 3.9 times as long as wide. Hind tibia weakly widened to apex, 7.9 times as long as its maximum sub-apical width, 0.9 times as long as hind tarsus. First segment of hind tarsus 1.9 times as long as second segment.

*Wings*. Length of fore wing 2.5 times its maximum width. Vein r1 present. Radial cell reaching to apex of wing, 4.4 times as long as its maximum width. Nervulus weakly postfurcal. Brachial cell closed, short, widened apically, 2.5 times as long as its maximum width. Hind wing 5.3 times as long as its maximum width.

*Metasoma*. Distinctly compressed. First tergite weakly widened towards apex, 1.8 times as long as its apical width, entirely finely and sparsely striate-rugose in apical half. Ovipositor 1.6 times as long as first tergite, distinctly shorter than metasoma, 1.1 times as long as hind femur.

*Colour*. Body and legs brown to dark brown. Wings hyaline. Pterostigma brown.

Male. Body 1.7–1.8 mm; fore wing 2.0–2.1 mm. Antenna 16–20-segmented. First flagellar segment 3.3 times as long as wide; second segment twice as long as wide. Third to seventeenth flagellar segments 1.9–2.0 times as long as their width, eighteenth segment 3.0 times as long as wide. Otherwise similar to female.

**Figures 25–30.**
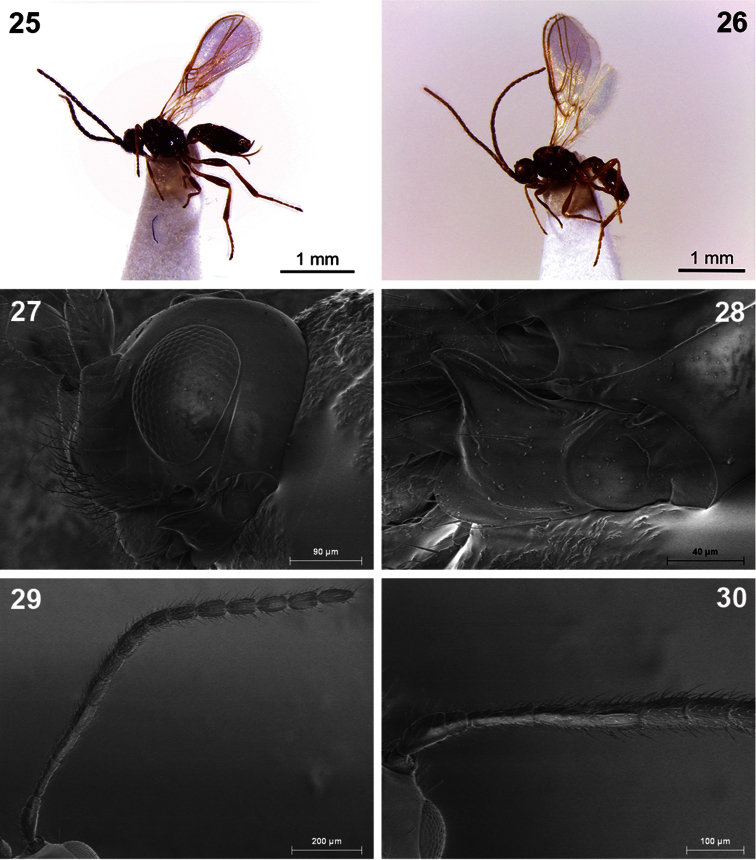
*Dinotrema lagunasense* sp. n. (**25, 27–30** female; **26** male). **25–26** Habitus, lateral view **27** Head, lateral view **28** Mandible **29** Antenna **30** Basal segments of antenna.

**Figures 31–36.**
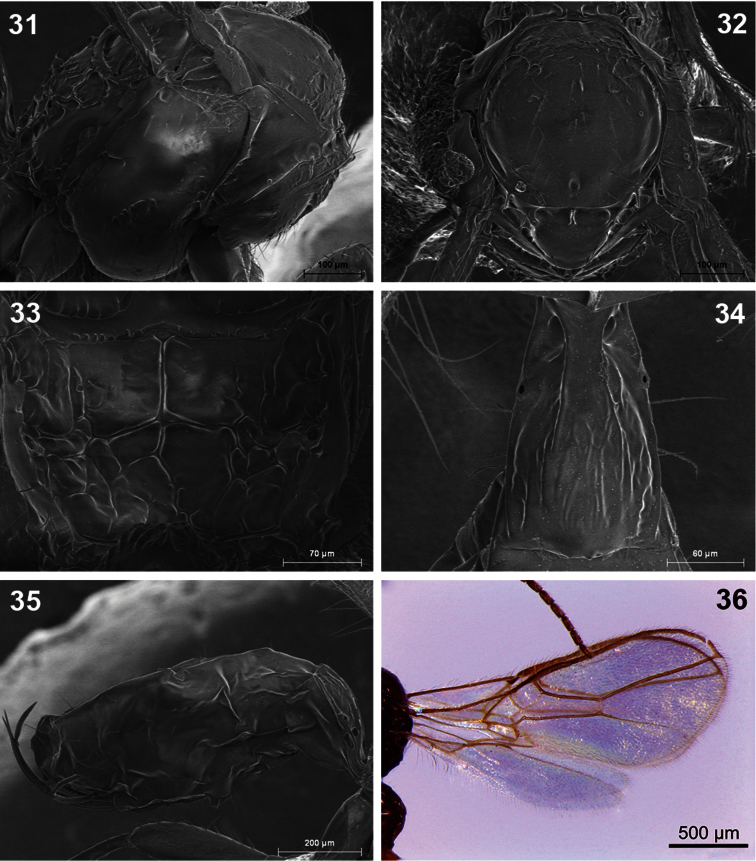
*Dinotrema lagunasense* sp. n. (female).**31** Mesosoma **32** Mesonotum **33** Propodeum **34** First metasomal tergite **35** Metasoma and ovipositor **36** Fore and hind wings.

#### Etymology.

The name is referring to the type locality of the new species, Natural Park of Las Lagunas de la Mata-Torrevieja in Alicante (Spain).

### Dinotrema pilarae

Peris-Felipo sp. n.

urn:lsid:zoobank.org:act:7E633024-040D-4300-BBDB-1C0FE75C41EE

http://species-id.net/wiki/Dinotrema_pilarae

Figs 37 –48

#### Type material.

Holotype: 1 female (ENV), “Spain, Alicante Province, Alcoi, Natural Park of Carrascal de La Font Roja, 13.i.2005, F.J. Peris-Felipo”. Paratype: 1 female (ENV), same label as holotype but 02.i.2007.

#### Diagnosis.

This new species resembles *Dinotrema nervosum* (Haliday) and *Dinotrema lineolum* (Thomson). *Dinotrema pilarae* sp. n. differs from *Dinotrema nervosum* in having the mandible 1.2 times as long as wide (1.5 times in *Dinotrema nervosum*), middle flagellar segments 1.9–2.0 times as long as their width (1.5 times in *Dinotrema nervosum*), precoxal sulcus reaching anterior margin of mesopleuron (not reaching in *Dinotrema nervosum*), and ovipositor shorter than metasoma (longer in *Dinotrema nervosum*). New species also differs from *Dinotrema lineolum* in having the mandible 1.2 times as long as wide (1.7 times in *Dinotrema nervosum*), first flagellar segment 3.2 times as long as wide (2.5 times in *Dinotrema lineolum*), middle flagellar segments 1.9–2.0 times as long as their width (1.5 times in *Dinotrema lineolum*), first metasomal tergite 1.8 times as long as its apical width (1.5 times in *Dinotrema lineolum*), and precoxal sulcus reaching anterior margin of mesopleuron (not reaching in *Dinotrema lineolum*).

#### Description.

Holotype, female, length of body 2.2 mm; fore wing 2.3 mm.

*Head*. In dorsal view, 1.8 times as wide as median length, 1.5 times as wide as mesoscutum, smooth, with rounded temples behind eye. Eye in lateral view 1.5 times as high as wide and as wide as temple. POL 2.6 times OD; OOL 3.4 times OD. Face 1.8 times as wide as high; inner margins of eyes subparallel. Clypeus 3.0 times as wide as high, slightly curved ventrally. Paraclypeal fovea short, not reaching the middle distance between clypeus and eye. Mandible widened towards apex, 1.2 times as long as its maximum width. Upper tooth of mandible longer than middle tooth and wider than lower tooth; middle tooth wide basally and narrowed towards apex, weakly rounded apically; lower tooth short, rounded apically. Antenna thick, 19-segmented. Scape twice as long as pedicel. First flagellar segment 3.2 times as long as its apical width, 1.1 times as long as second segment; second segment 2.1 times as long as its maximum width. Third to fifth flagellar segments 2.1–2.3 times as long as their width, sixth to seventeenth flagellar segments 1.9–2.0 times as long as their width.

*Mesosoma*. In lateral view, as long as high. Mesoscutum as long as maximum width. Notauli largely absent. Mesoscutal pit present and oval. Prescutellar depression smooth, with lateral carinae. Precoxal sulcus present, reaching anterior margin of mesopleuron. Posterior mesopleural furrow crenulate in lower half. Propodeum sculptured, with complete median longitudinal carina crossing from anterior to posterior margins, with emerging lateral carina from third apical part reaching propodeal margins near spiracles place delineated large areola. Propodeal spiracles relatively small.

*Legs*. Hind femur 4.1 times as long as wide. Hind tibia weakly widened to apex, 8.3 times as long as its maximum subapical width, as long as hind tarsus. First segment of hind tarsus 2.1 times as long as second segment.

*Wings*. Length of fore wing 2.4 times its maximum width. Vein r1 present. Radial cell reaching to apex of wing, 4.3 times as long as its maximum width. Nervulus weakly postfurcal. Brachial cell closed, short, widened apically, 2.7 times as long as its maximum width. Hind wing 5.1 times as long as its maximum width.

*Metasoma*. Distinctly compressed. First tergite weakly widened towards apex, 1.8 times as long as its apical width, striate in apical half on wide lateral areas, but almost smooth on narrow median area. Ovipositor 1.8 times as long as first tergite, shorter than metasoma, 1.1 times as long as hind femur.

*Colour*. Body and legs brown to dark brown. Wings hyaline. Pterostigma brown.

*Male*. Unknown.

**Figures 37–42.**
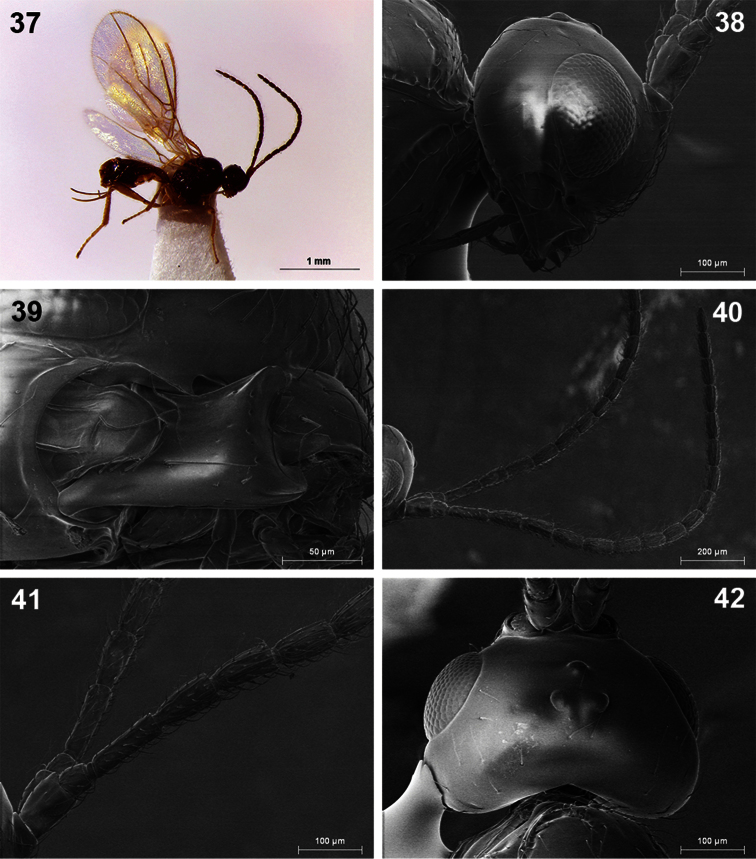
*Dinotrema pilarae* sp. n. (female). **37** Habitus, lateral view **38** Head, lateral view **39** Mandible **40** Antenna **41** Basal segments of antenna **42** Head, dorsal view.

**Figures 43–48.**
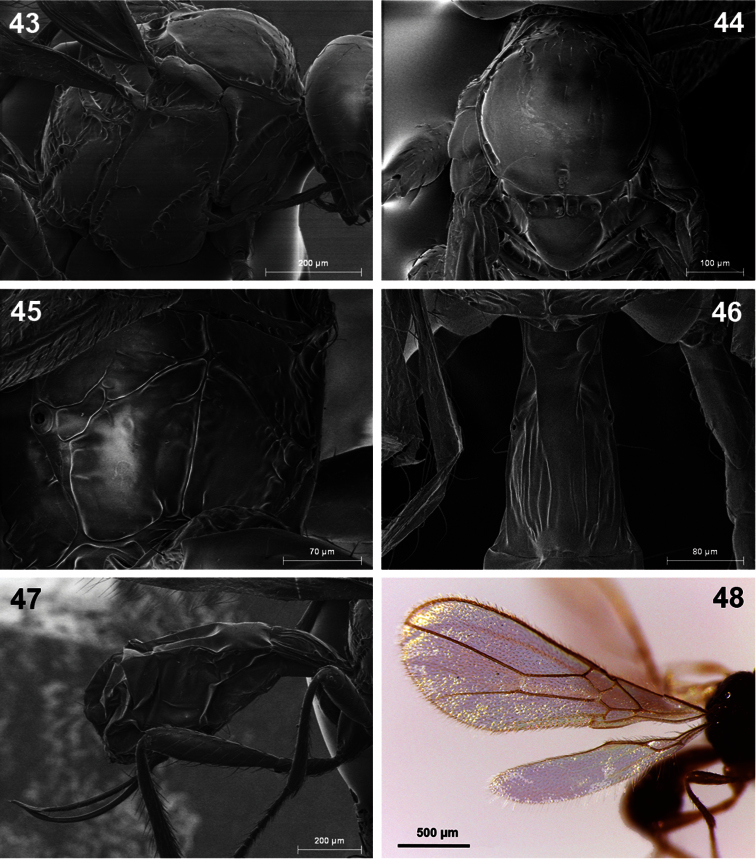
*Dinotrema pilarae* sp. n. (female).**43** Mesosoma **44** Mesonotum **45** Propodeum **46** First metasomal tergite **47** Metasoma and ovipositor **48** Fore and hind wings.

#### Etymology.

Named in honour of Dr. Pilar Gonzalez Funes, retired teacher in University of Valencia and who gave me the first opportunity to study entomology.

### Dinotrema robertoi

Peris-Felipo sp. n.

urn:lsid:zoobank.org:act:28088352-380E-45AD-99FC-6D22B795BC40

http://species-id.net/wiki/Dinotrema_robertoi

Figs 49 –60

#### Type material.

Holotype: 1 female (ENV), “Spain, Castellon Province, Pobla de Benifassà, Natural Park of Tinença de Benifassà, 22.vii.2004, F.J. Peris-Felipo”. Paratypes: 1 female (ENV), same label as holotype but 05.viii.2004; 1 female (ENV), same label as holotype but 16.ix.2004.

#### Diagnosis.

This new species resembles *Dinotrema compressum* (Haliday) (comb. n.), but differs in having hind femur 3.5 times as long as its maximum width (4.0 times in *Dinotrema compressum*), first metasomal tergite 2.5 times as long as its apical width and smooth in apical half (2.3 times and striate in apical half in *Dinotrema compressum*), prescutellar depression rectangular and with lateral carinae (subsquare and without lateral carinae in *Dinotrema compressum)*, and middle tooth short and apically rounded (large and pointed in *Dinotrema compressum*).

#### Description.

Holotype, female. length of body 1.0 mm; fore wing 1.1 mm.

*Head*. In dorsal view, 1.5 times as wide as median length, 1.5 times as wide as mesoscutum, smooth, with rounded temples behind eye. Eye in lateral view 1.3 times as high as wide and 0.8 times as wide as temple. POL 3.6 times OD; OOL 4.7 times OD. Face 1.2 times as wide as high; inner margins of eyes subparallel. Clypeus 3.7 times as wide as high, slightly curved ventrally. Paraclypeal fovea short, not reaching the middle distance between clypeus and eye. Mandible widened towards apex, 0.95 times as long as its maximum width. Upper tooth of mandible larger than middle tooth and distinctly wider than lower tooth; middle tooth short, wide basally, distinctly narrowed towards apex, weakly rounded apically; lower tooth rounded apically. Antennae thick, 12-segmented. Scape 1.6 times as long as pedicel. First flagellar segment 2.6 times as long as its apical width, as long as second segment; second segment 2.1 times as long as its maximum width. Third to ninth flagellar segments 1.6 times as long as their width, tenth flagellar segment twice as long as its width.

*Mesosoma*. In lateral view, 1.3 times as long as high. Mesoscutum as long as maximum width. Notauli largely absent. Mesoscutal pit absent. Prescutellar depression smooth, without lateral carinae. Precoxal sulcus present, not reaching anterior and posterior margins of mesopleuron. Posterior mesopleural furrow slightly crenulate below. Propodeum sculptured, with short median longitudinal carina, with emerging lateral carinae reaching propodeal edges. Propodeal spiracles relatively small.

*Legs*. Hind femur 3.5 times as long as wide. Hind tibia weakly widened to apex, about 7.9 times as long as its maximum sub-apical width, 0.9 times as long as hind tarsus. First segment of hind tarsus 1.7 times as long as second segment.

*Wings*. Length of fore wing 2.6 times its maximum width. Vein r1 present. Radial cell reaching to apex of wing, 4.0 times as long as its maximum width. Nervulus weakly postfurcal. Brachial cell closed, short, widened apically, 1.5 times as long as its maximum width. Hind wing 7.2 times as long as its maximum width.

*Metasoma*. Distinctly compressed. First tergite weakly widened towards apex, 2.5 times as long as its apical width, smooth. Ovipositor 0.7 times as long as first tergite, distinctly shorter than metasoma, 0.4 times as long as hind femur.

*Colour*. Body and legs dark brown. Wings hyaline. Pterostigma brown.

*Male*. Unknown.

**Figures 49–54.**
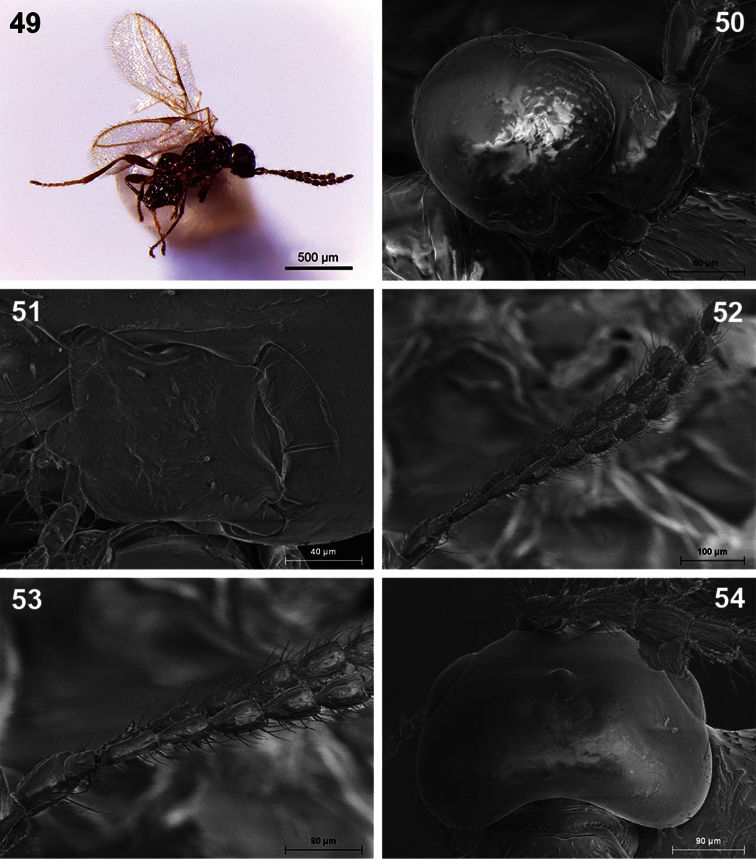
*Dinotrema robertoi* sp. n. (female). **49** Habitus, lateral view **50** Head, lateral view **51** Mandible **52** Antenna **53** Basal segments of antenna **54** Head, dorsal view.

**Figures 55–60.**
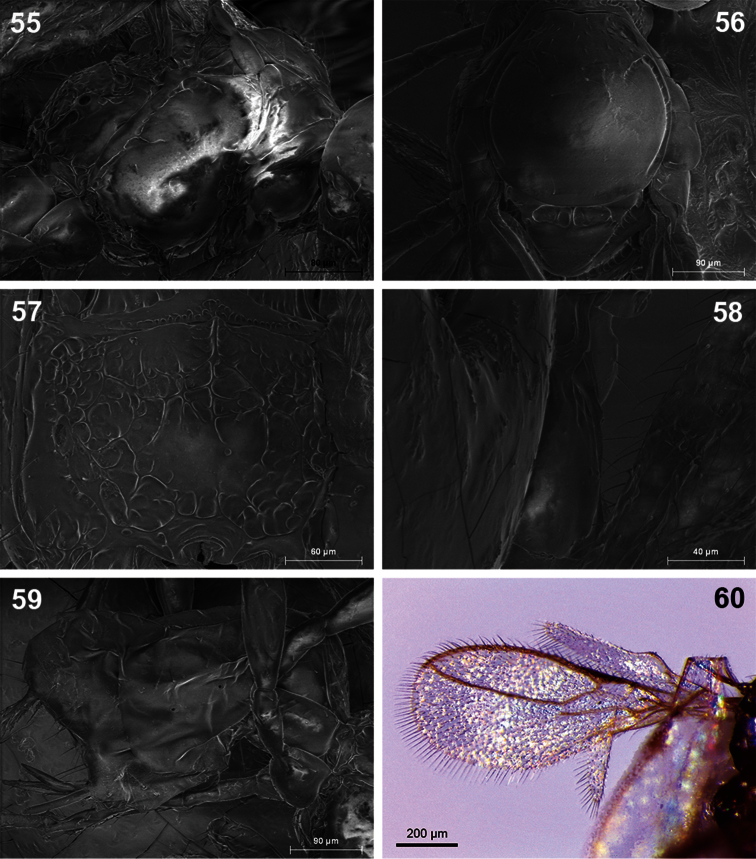
*Dinotrema robertoi* sp. n. (female).**55** Mesosoma **56** Mesonotum **57** Propodeum **58** First metasomal tergite **59** Metasoma and ovipositor **60** Fore wing.

#### Etymology.

Named in honour of Roberto Peris for his help, support and patience during my work on PhD thesis.

### Dinotrema teresae

Peris-Felipo sp. n.

urn:lsid:zoobank.org:act:7CBC1FE8-B193-4B3F-8256-1735CE0ACF52

http://species-id.net/wiki/Dinotrema_teresae

Figs 61 –72

#### Type material.

Holotype: 1 femal (ENV), “Spain, Castellon Province, Pobla de Benifassà, Natural Park of Tinença de Benifassà, 30.iv.2007, F.J. Peris-Felipo”. Paratype: 1 female (ENV), same label as holotype but 24.iv.2006.

#### Diagnosis.

This new species resembles *Dinotrema eumandibulatum* (Fischer) and *Dinotrema dentipraesens* (Fischer). New species differs from *Dinotrema eumandibulatum* in having the mandible 1.05 times as long as wide (0.8 times in *Dinotrema eumandibulatum*), first flagellar segment 2.85 times as long as wide (3.3 times in *Dinotrema eumandibulatum*), hind femur 4.0 times as long as its maximum width (3.5 times in *Dinotrema eumandibulatum*), and first metasomal tergite striated in apical half (almost smooth in *Dinotrema eumandibulatum*). *Dinotrema teresae* sp. n. differs from *Dinotrema dentipraesens* in having the first flagellar segment 2.85 times as long as wide (3.25 times in *Dinotrema dentipraesens*), middle flagellar segments 1.50–1.65 times as long as their width (2.25 times in *Dinotrema dentipraesens*), mesoscutal pit oval (rounded in *Dinotrema dentipraesens*), and first metasomal tergite striated in apical half (smooth in *Dinotrema dentipraesens*).

#### Description.

Holotype, female, length of body 1.7 mm; fore wing 2.1 mm.

*Head*. In dorsal view, 1.65 times as wide as median length, 1.55 times as wide as mesoscutum, smooth, with rounded temples behind eye. Eye in lateral view 1.65 times as high as wide and 0.9 times as wide as temple. POL 3.75 times OD; OOL 4.0 times OD. Face 1.95 times as wide as high; inner margins of eyes subparallel. Clypeus 3.0 times as wide as high, slightly curved ventrally. Paraclypeal fovea elongated, reaching the middle distance between clypeus and eye. Mandible widened towards apex, 1.05 times as long as its maximum width. Upper tooth of mandible large, but weakly shorter than middle tooth; middle tooth the longest, wide basally and narrowed towards apex, weakly rounded apically; lower tooth short and wider than upper tooth, rounded apically. Antennae thick, more than 13-segmented (apical segments missing). Scape 2.45 times as long as pedicel. First flagellar segment 2.85 times as long as its apical width, 1.15 times as long as second segment; second segment twice as long as its maximum width. Third to tenth flagellar segments 1.50–1.65 times as long as their width.

*Mesosoma*. In lateral view, 1.05 times as long as high. Mesoscutum 0.95 times as long as maximum width. Notauli largely absent. Mesoscutal pit present and elongated. Prescutellar depression smooth, without lateral carinae. Precoxal sulcus present, not reaching anterior and posterior margins of mesopleuron. Posterior mesopleural furrow smooth. Propodeum sculptured, with median longitudinal carina crossing from anterior to posterior margins, medially with emerging carinae and rugae reaching propodeal margins. Propodeal spiracles relatively small.

*Legs*. Hind femur 4.0 times as long as wide. Hind tibia weakly widened to apex, about 8.8 times as long as its maximum subapical width, 0.95 times as long as hind tarsus. First segment of hind tarsus 0.9 times as long as second segment.

*Wings*. Length of fore wing 2.8 times its maximum width. Vein r1 present. Radial cell reaching to apex of wing, 4.5 times as long as its maximum width. Nervulus weakly postfurcal. Brachial cell closed, short, widened apically, 2.3 times as long as its maximum width. Hind wing 6.5 times as long as its maximum width.

*Metasoma*. Distinctly compressed. First tergite weakly widened towards apex, 2.2 times as long as its apical width, in apical half distinctly striate with rugulosity. Ovipositor 0.5 times as long as first tergite, distinctly shorter than metasoma, 0.4 times as long as hind femur.

*Colour*. Body and legs dark brown. Wings hyaline. Pterostigma brown.

*Male*. Unknown.

**Figures 61–66.**
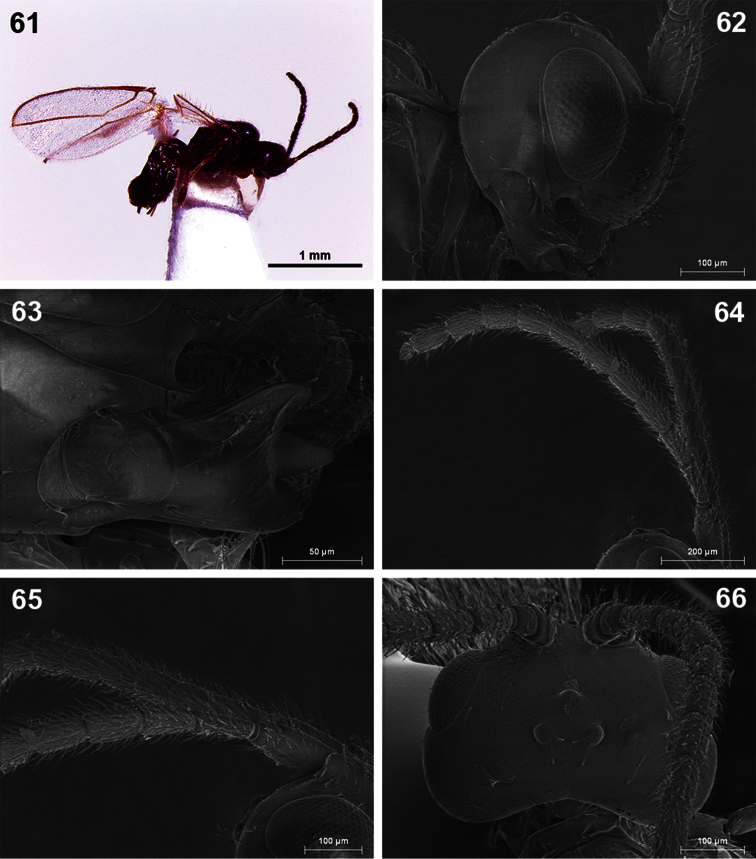
*Dinotrema teresae* sp. n. (female). **61** Habitus, lateral view **62** Head, lateral view **63** Mandible **64** Antenna **65** Basal segments of antenna **66** Head, dorsal view.

**Figures 67–72.**
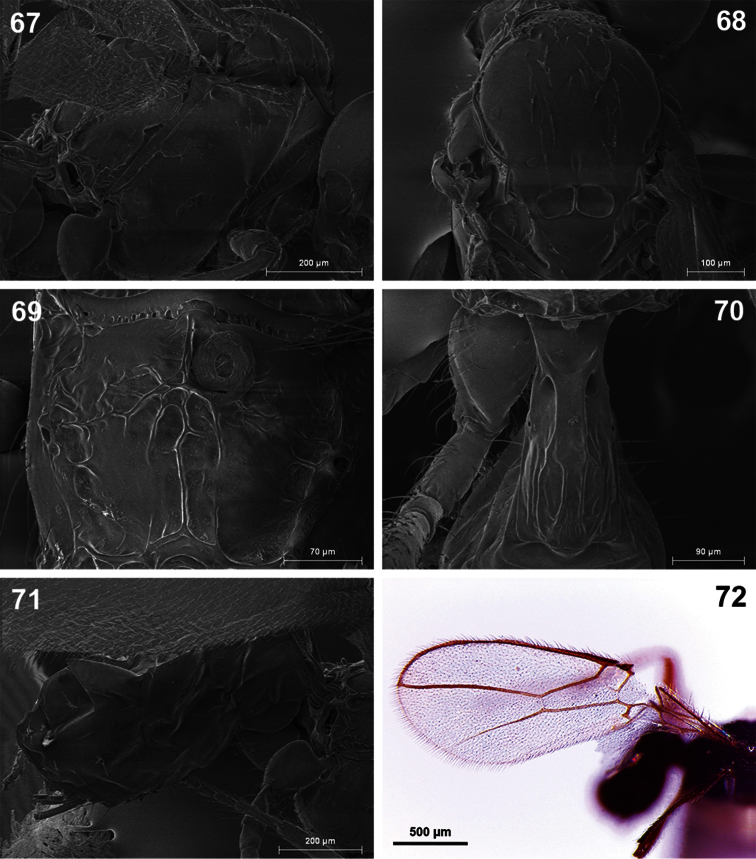
*Dinotrema teresae* sp. n. (female) **67** Mesosoma **68** Mesonotum **69** Propodeum **70** First metasomal tergite **71** Metasoma and ovipositor **72** Fore wing.

#### Etymology.

Named in honour of Dr Maria Teresa Oltra, for her contributions to the study of Braconidae, her great help and support.

### Dinotrema tinencaense

Peris-Felipo sp. n.

urn:lsid:zoobank.org:act:AAED42E0-2909-4B84-8D09-91CFE581AFD6

http://species-id.net/wiki/Dinotrema_tinencaense

Figs 73 –84

#### Type material.

Holotype: 1 female (ENV), “Spain, Castellon Province, Pobla de Benifassà, Natural Park of Tinença de Benifassà, 10.vi.2004, F.J. Peris-Felipo”. Paratypes: 1 female (ENV), same label as holotype but 05.viii.2004; 1 female (ENV), same label as holotype but 27.ix.2004; 1 female (ENV), same label as holotype but 15.vii.2004; 1 female (ENV), same label as holotype but 22.vii.2004; 2 females (ENV), same label as holotype but 13.vi.2005; 1 female (ZISP), same label as holotype but 04.vii.2005; 1 female (ZISP), same label as holotype but 04.vii.2005.

#### Diagnosis.

This new species resembles *Dinotrema arenarium* (Tobias) and *Dinotrema falsificum* (Stelfox et Graham). New species differs from *Dinotrema arenarium* in having the mandible 1.1 times as long as wide (1.4 times in *Dinotrema arenarium*), first flagellar segment 2.5 times as long as its width (4.0 times in *Dinotrema arenarium*), middle flagellar segments 1.5–1.6 times as long as their width (twice in *Dinotrema arenarium*), precoxal sulcus not reaching anterior and posterior margins of mesopleuron (reaching in *Dinotrema arenarium*), and prescutellar depression with lateral carinae (without lateral carinae in *Dinotrema arenarium*). On the other hand, *Dinotrema tinencaense* sp. n. differs from *Dinotrema falsificum* in having the mandible 1.1 times as long as wide (twice in *Dinotrema falsificum*), first flagellar segment 2.5 times as long as wide (3.5 times in *Dinotrema falsificum*), middle flagellar segments 1.5–1.6 times as long as their width (twice in *Dinotrema falsificum*), hind femur 3.6–3.7 times as long as its maximum width (5.0 times in *Dinotrema falsificum*), first metasomal tergite 1.4 times as long as its apical width (2.2 times in *Dinotrema falsificum*), and ovipositor distinctly shorter than metasoma (as long as metasoma in *Dinotrema falsificum*).

#### Description.

Holotype, female, length of body 1.3 mm; fore wing 1.3 mm.

*Head*. In dorsal view, 1.45 times as wide as median length, 1.5 times as wide as mesoscutum, smooth, with rounded temples behind eye. Eye in lateral view 1.65 times as high as wide and 0.7 times as wide as temple. POL 3.3 times OD; OOL 4.05 times OD. Face twice as wide as high; inner margins of eyes subparallel. Clypeus 2.8 times as wide as high, slightly curved ventrally. Paraclypeal fovea short, not reaching middle distance between clypeus and eye. Mandible widened towards apex, 1.1 times as long as its maximum width. Upper tooth of mandible longer than middle tooth and wider than lower tooth; middle tooth wide basally and strongly narrowed towards apex, weakly rounded apically; lower tooth short, rounded apically. Antennae thick, 14-segmented. Scape 1.75 times as long as pedicel. First flagellar segment 2.5 times as long as its apical width, as long as second segment; second segment twice as long as its maximum width. Third to eleventh flagellar segments 1.5–1.6 times as long as their width, twelfth flagellar segment twice as long as its width.

*Mesosoma*. In lateral view, 1.15 times as long as high. Mesoscutum 0.9 times as long as maximum width. Notauli largely absent. Mesoscutal pit absent. Prescutellar depression smooth, with lateral carinae. Precoxal sulcus present, not reaching anterior and posterior margins of mesopleuron. Posterior mesopleural furrow crenulate in lover half. Propodeum sculptured, with short median longitudinal carina diverging in two long carinae, with numerous carinae and rugae covered most part of propodeum and reaching its margins. Propodeal spiracles relatively small.

*Legs*. Hind femur 3.6 times as long as wide. Hind tibia weakly widened to apex, about 9.5 times as long as its maximum subapical width, as long as hind tarsus. First segment of hind tarsus 2.05 times as long as second segment.

*Wings*. Length of fore wing 2.75 times its maximum width. Vein r1 present. Radial cell reaching to apex of wing, 4.0 times as long as its maximum width. Nervulus weakly postfurcal. Brachial cell closed, short, distinctly widened apically, 1.8 times as long as its maximum width. Hind wing 6.5 times as long as its maximum width.

*Metasoma*. Distinctly compressed. First tergite weakly widened towards apex, 1.4 times as long as its apical width, mainly distinctly striate, smooth on narrow median area. Ovipositor 1.25 times as long as first tergite, distinctly shorter than metasoma, 0.65 times as long as hind femur.

*Colour*. Body and legs brown to dark brown. Wings hyaline. Pterostigma brown.

*Male*. Unknown.

**Figures 73–78.**
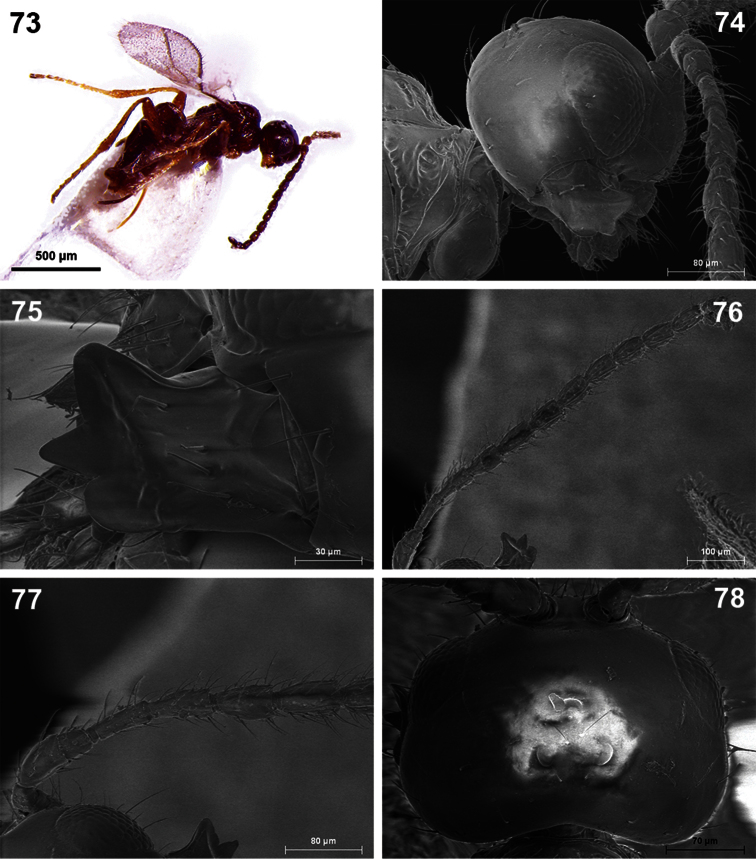
*Dinotrema tinencaense* sp. n. (female). **73** Habitus, lateral view **74** Head, lateral view **75** Mandible **76** Antenna **77** Basal segments of antenna **78** Head, dorsal view.

**Figures 79–84.**
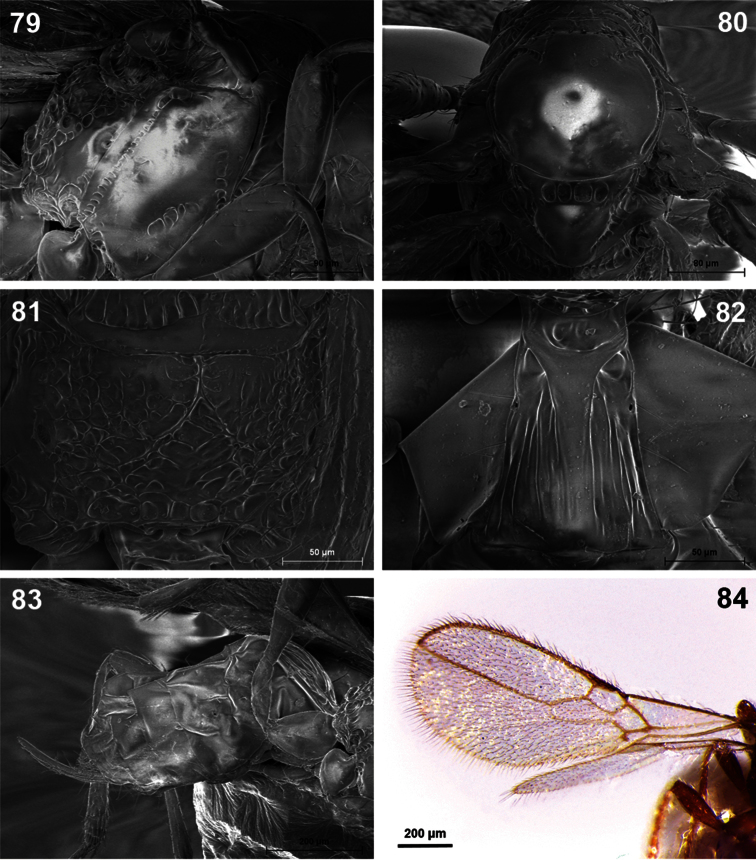
*Dinotrema tinencaense* sp. n. (female).**79** Mesosoma **80** Mesonotum **81** Propodeum **82** First metasomal tergite **83** Metasoma and ovipositor **84** Fore and hind wings.

#### Etymology.

The name is referring to the type locality of the new species, Natural Park of La Tinença de Benifassà in Castellon (Spain).

### Dinotrema torreviejaense

Peris-Felipo sp. n.

urn:lsid:zoobank.org:act:7840356B-3479-4ED5-9A39-0063215FB671

http://species-id.net/wiki/Dinotrema_torreviejaense

Figs 85 –96

#### Type material.

Holotype: 1 female (ENV), “Spain, Alicante Province, Torrevieja, Natural Park of Lagunas de La Mata-Torrevieja, 04.iv.2004, F.J. Peris-Felipo”. Paratype: 1 female (ENV), same label as holotype but 09.v.2006.

#### Diagnosis.

This new species resembles *Dinotrema lagunasense* Peris-Felipo sp. n., *Dinotrema adventum* (Fischer) (comb. n.), *Dinotrema sternaulicum* (Fischer) and *Dinotrema necrophilum* (Hedqvist). New species differs from *Dinotrema lagunasense* in having the first metasomal tergite 2.3 times as long as its apical width and mainly smooth (1.8 times and finely striate in apical half in *Dinotrema lagunasense*), hind femur 3.6 times as long as its maximum width (3.9 times in *Dinotrema lagunasense*), and mesoscutal pit elongate-oval (rounded in *Dinotrema lagunasense*). On the other hand, *Dinotrema torreviejaense* sp. n. differs from *Dinotrema adventum* in having the mandible 0.9 times as long as wide (1.45 times in *Dinotrema adventum*), first flagellar segment 2.75 times as long as wide (twice in *Dinotrema adventum*), hind femur 3.6 times as long as its maximum width (4.0 times in *Dinotrema adventum*), and first metasomal tergite 2.3 times as long as its apical width (twice in *Dinotrema adventum*). Also, new species differs from *Dinotrema sternaulicum* in having the mandible 0.9 times as long as wide (1.4 times in *Dinotrema sternaulicum*), first flagellar segment 2.75 times as long as wide (3.0 times in *Dinotrema sternaulicum*), middle flagellar segments 1.8–1.9 times as long as their width (1.5 times in *Dinotrema sternaulicum*), and first metasomal tergite 2.3 times as long as its apical width (1.75 times in *Dinotrema sternaulicum*). Finally, *Dinotrema torreviejaense* sp. n. differs from *Dinotrema necrophilum* in having the mandible 0.9 times as long as wide (1.45 times in *Dinotrema necrophilum*), middle flagellar segments 1.8–1.9 times as long as their width (1.3 times in *Dinotrema necrophilum*), hind femur 3.6 times as long as its maximum width (4.1 times in *Dinotrema necrophilum*), and first metasomal tergite 2.3 times as long as its apical width (1.6 times in *Dinotrema necrophilum*).

#### Description.

Holotype, female, length of body 1.7 mm; fore wing 1.7 mm.

*Head*. In dorsal view, 1.9 times as wide as median length, 1.5 times as wide as mesoscutum, smooth, with rounded temples behind eye. Eye in lateral view 1.75 times as high as wide and 0.75 times as wide as temple. POL 3.15 times OD; OOL 3.15 times OD. Face 1.4 times as wide as high; inner margins of eyes subparallel. Clypeus 3.0 times as wide as high, slightly curved ventrally. Paraclypeal fovea elongate, reaching middle distance between clypeus and eye. Mandible strongly widened, 0.9 times as long as its maximum width. Upper tooth of mandible long, but weakly shorter than middle tooth; middle tooth the longest, wide basally and narrowed towards apex, weakly rounded apically; lower tooth short and wide, wider than upper tooth, rounded. Antennae thick, more than 13-segmented (apical segments missing). Scape twice as long as pedicel. First flagellar segment 2.75 times as long as its apical width, 1.1 times as long as second segment; second segment twice as long as its maximum width. Third to eleventh flagellar segments 1.8–1.9 times as long as their width.

*Mesosoma*. In lateral view, 1.1 times as long as high. Mesoscutum 0.95 times as long as maximum width. Notauli largely absent. Mesoscutal pit present and elongate-oval. Prescutellar depression smooth, without lateral carinae. Precoxal sulcus present, not reaching anterior and posterior margin of mesopleuron. Posterior mesopleural furrow smooth. Propodeum sculptured, with short medio-basal carina and pentagonal areola, its lateral carinae reaching propodeal edges, with sparse rugae laterally. Propodeal spiracles relatively small.

*Legs*. Hind femur 3.6 times as long as wide. Hind tibia weakly widened to apex, 8.3 times as long as its maximum sub-apical width, 1.2 times as long as hind tarsus. First segment of hind tarsus 2.1 times as long as second segment.

*Wings*. Length of fore wing 2.6 times its maximum width. Vein r1 present. Radial cell reaching to apex of wing, 4.4 times as long as its maximum width. Nervulus weakly postfurcal. Brachial cell closed, short, widened apically, about 2.5 times as long as its maximum width. Hind wing about 6.0 times as long as its maximum width.

*Metasoma*. Distinctly compressed. First tergite weakly widened towards apex, 2.3 times as long as its apical width, smooth on wide area, partly finely sculptured in apical third. Ovipositor 1.9 times as long as first tergite, shorter than metasoma, 1.4 times as long as hind femur.

*Colour*. Body and legs brown to dark brown. Wings hyaline. Pterostigma brown.

*Male*. Unknown.

**Figures 85–90.**
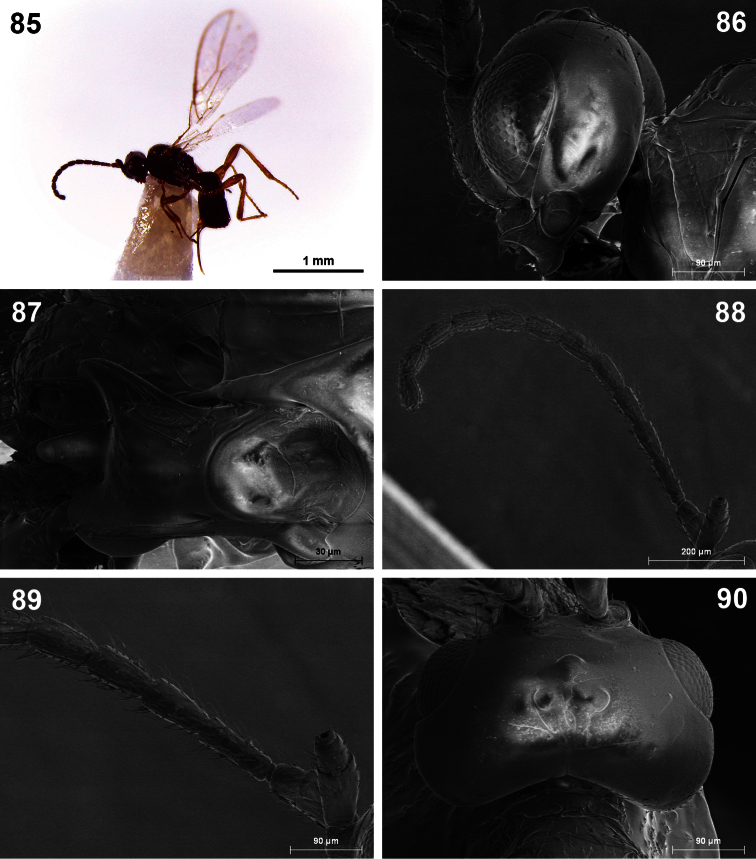
*Dinotrema torreviejaense* sp. n. (female). **85** Habitus, lateral view **86** Head, lateral view **87** Mandible **88** Antenna **89** Basal segments of antenna **90** Head, dorsal view.

**Figures 91–96.**
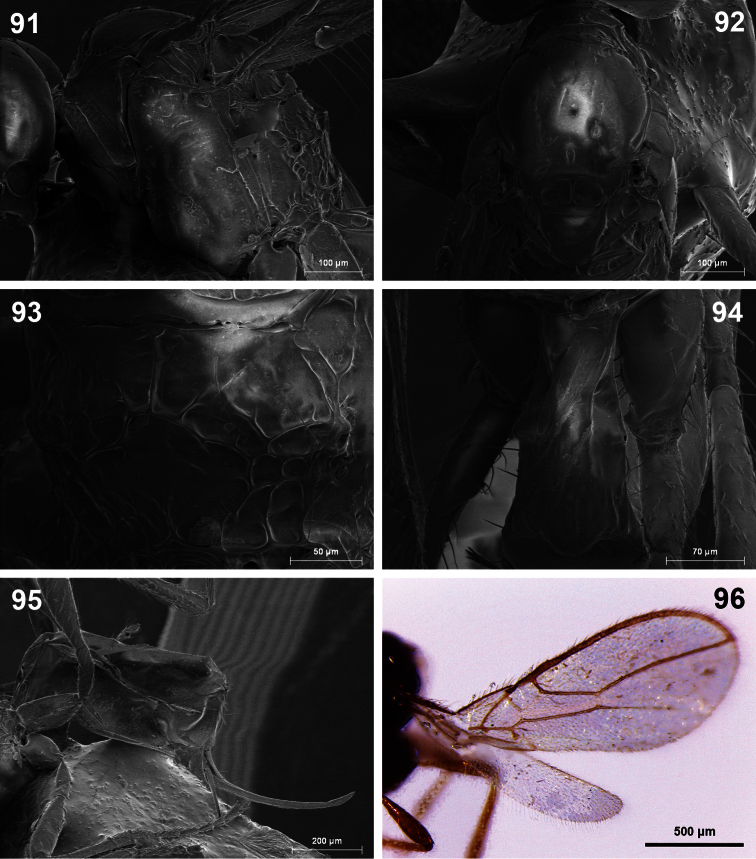
*Dinotrema torreviejaense* sp. n. (female).**91** Mesosoma **92** Mesonotum **93** Propodeum **94** First metasomal tergite **95** Metasoma and ovipositor **96** Fore and hind wings.

#### Etymology.

The name is referring to the type locality of the new species, Natural Park of Las Lagunas de la Mata-Torrevieja in Alicante (Spain).

## Supplementary Material

XML Treatment for Dinotrema amparoae

XML Treatment for Dinotrema benifassaense

XML Treatment for Dinotrema lagunasense

XML Treatment for Dinotrema pilarae

XML Treatment for Dinotrema robertoi

XML Treatment for Dinotrema teresae

XML Treatment for Dinotrema tinencaense

XML Treatment for Dinotrema torreviejaense
